# Cadmium Induces Liver Cell Apoptosis through Caspase-3A Activation in Purse Red Common Carp (*Cyprinus carpio*)

**DOI:** 10.1371/journal.pone.0083423

**Published:** 2013-12-12

**Authors:** Dian Gao, Zhen’e Xu, Panpan Qiao, Shen Liu, Li Zhang, Penghui He, Xiaoyan Zhang, Yannan Wang, Weiping Min

**Affiliations:** 1 Medical College of Nanchang University, Nanchang, PR China; 2 Institute of Immunotherapy, Nanchang University, Nanchang, PR China; 3 Jiangxi Academy of Medical Sciences, Nanchang, PR China; Kaohsiung Chang Gung Memorial Hospital, Taiwan

## Abstract

Caspase-3, the essential effector caspase, plays a pivotal role during caspase-dependent apoptosis. In this study, we isolated and characterized caspase-3A gene from common carp. The common carp caspase-3A comprising 273 amino acids showed 71.8% sequence similarity and 59.3% sequence identity to human caspase-3. It exhibited an evolutionarily conserved structure of mammalian caspase-3 genes, including a pro-domain, a large subunit, a small subunit and other motifs such as the pentapeptide active-site motif (QACRG) and the putative cleavage sites at the aspartic acids. Phylogenetic analysis demonstrated that common carp caspase-3A formed a clade with cyprinid fish caspase-3. To assess whether caspase-3A is involved in cadmium (Cd)-induced cell apoptosis in common carp, a Cd exposure experiment was performed. TUNEL analysis showed that Cd triggered liver cell apoptosis; caspase-3A activity was markedly increased; its proenzyme level was significantly decreased, and the levels of its cleaved forms were markedly increased. However, real-time quantitative PCR analysis revealed that the mRNA transcript level of caspase-3A was not significantly elevated. Immunoreactivities were observed in the cytoplasm of hepatocytes by immunohistochemical detection. The findings indicates that Cd can trigger liver cell apoptosis through the activation of caspase-3A. Caspase-3A may play an essential role in Cd-induced apoptosis.

## Introduction

Apoptosis, or programmed cell death, plays a critical role in the survival of multicellular organisms by getting rid of damaged or infected cells that may interfere with normal function [1].It can be regulated by many modulators, including some ions (e.g. calcium), genes (e.g. c-myc, Bcl-2/Bax and Fas), proteins (e.g. p53, caspases, IAPs) and even organelles (e.g. mitochondria, endoplasmic reticulum) [2]. Among these regulators, members of the caspase family of aspartic acid-directed cysteine proteases, lead to the loss of cellular structure and function and eventually result in apoptotic cell death [3], [4]. In mammalian cells, the caspase family comprises at least 14 enzymes, which can be mainly divided into two categories, initiator caspases and executioner caspases depending on where they function in the apoptotic cascade [5]. Initiator caspases include caspase-2, -8, -9 and -10. Once activated, they cleave and activate the executioner caspases, which consist of caspases-3, to a lesser extent caspase-6 and -7. The activated executioner caspases then cleave their respective substrates to cause demolition of the cell [6]. Briefly, the caspase activation is regulated through either extrinsic pathway (death receptor pathway) or intrinsic pathway (mitochondrial pathway) [7]. Both pathways converge on caspase-3 and subsequently on other proteases and nucleases that drive the terminal events of apoptosis [8], [9].

Apoptosis can be induced by many different stimuli such as ultraviolet (UV) irradiation [10], chemotherapy agents [11], infection by pathogens [12], [13], polychlorinated biphenyls (PCBs) [14], polycyclic aromatic hydrocarbons (PAHs) [15], insecticides [16] and heavy metals [17], [18]. Among these factors, heavy metals such as Cd play crucial roles in the process of apoptosis induction, and may thus tip the balance of cellular homeostasis towards an increased cellular mortality [19]. As a widespread environmental pollutant, Cd has multiple effects on cells, affecting essential cellular processes such as cell division, proliferation, differentiation and apoptosis [20]. Cd triggers cell apoptosis, both in vitro [17], [21] and in vivo [22], [23] in several models, but the mechanisms remain controversial. Commonly, Cd can induce apoptosis via a caspase-dependent pathway or a caspase-independent pathway based on the different Cd exposure conditions [17], [24]–[26].

Similar to the findings reported for mammalian models, a few studies about fish tissues and cells have indicated the occurrence of apoptosis upon Cd exposure. In rainbow trout (*Oncorhynchus mykiss*) cell lines, Cd exposure promotes the elevation of caspase-3 activity [27]. In larval topsmelt (*Atherinops affinis*), Cd treatment increases apoptotic DNA fragmentation [28]. In zebrafish (*Danio rerio*), Cd contamination effects pro-apoptotic genes bax and *c-jun* to be up-regulated [29]. Generally, Cd-induced apoptosis correlates with caspase-dependent pathway in fish. As an effector caspase, caspase-3 is situated at a pivotal junction in the apoptotic pathways triggered both by the mitochondrial and the death receptor pathways [30]. In fish, It has been mainly identified and characterized in the following fish species: cinnamon clownfish (*Amphiprion melanopus*) [31], rock bream (*Oplegnathus fasciatus*) [32], European sea bass (*Dicentrarchus labrax*) [33], zebrafish [34], large yellow croaker (*Pseudosciaena crocea*) [35], Atlantic salmon (*Salmo salar*) [36], Nile tilapia (*Oreochromis niloticus*) [37] and Medaka (*Oryzias latipes*) [38]. Moreover, two isoforms of caspase-3 (A and B) have been identified in Atlantic salmon [36] and Medaka [38]. These caspase-3 homologs show similar and conserved immune-related functions between fish and mammals. They are involved in the process of apoptosis response to viral or bacterial challenge [32], [35].

Common carp is one of the major farmed freshwater species in the world [39]. It exhibits a strong tolerance to environmental stress and has become a widely used model species [40]. However, despite its great economic value and ecological importance in world aquaculture, innate immunological knowledge about common carp is relatively poor compared with other cultured fish such as rainbow trout, Atlantic salmon, European sea bass and Medaka. To date, only a few apoptotic genes such as tumor necrosis factor-α (TNF-α) [41] and recruitment domain protein (CARD) [42] have been completely identified and characterized. It is necessary to get a clear understanding of the immunological mechanisms against stimuli such as infection and heavy metal toxicity in common carp. In this study, to explore whether caspase-3 is involved in Cd-induced apoptosis in common carp, we isolated complementary DNA (cDNA) of caspase-3 from common carp and studied its roles in liver tissues upon Cd exposure.

## Materials and Methods

### Ethics Statement

All the procedures involving animals are in compliance with the care and use guidelines of experimental animals established by the Ministry of Agriculture of China. The study was approved by the ethics committee of Nanchang University (protocol number: NCU20121130).

### Cloning of caspase-3 from common carp

Total RNA was extracted using TRIzol reagent (Invitrogen, USA) from common carp liver according to the manufacturer’s instructions. A first-strand cDNA sequence was amplified using a SMART PCR cDNA Synthesis Kit (Clontech, USA) according to the manufacturer’s instructions. Degenerate primers (Csp3F, Csp3R) were designed to obtain caspase-3 gene based on the conserved region of caspase-3 sequences from zebrafish (GenBank accession no. NM_131877.3 and NM_001048066.1), white cloud mountain minnow (*Tanichthys albonubes*, GenBank no. GQ406344.1) and Medaka (GenBank accession no. NM_001104670.1 and NM_001104698.1). PCR cycling conditions were performed at 94°C for 5 min; 30 cycles at 94°C for 30 s, 55°C for 60 s, and 72°C for 60 s; followed by 1 cycle at 72°C for 10 min. To obtain the full cDNA sequence, The 3′ and 5′ ends were obtained by rapid amplification of cDNA ends (RACE) approaches, which were carried out using the gene-specific primers and adaptor primers (UPM) with the following PCR program: 1 cycle at 94°C for 5 min; 10 cycles at 94°C for 30 s, 60°C for 60 s, and 72°C for 60 s; 25 cycles at 94°C for 30 s, 55°C for 60 s, and 72°C for 60 s; followed by 1 cycle at 72°C for 10 min. All the purified fragments were then cloned and sequenced. The primers used in this study are listed in Table 1.

**Table 1:** Primers used for caspase-3A gene cloning and expression analysis.

Primer name	Nucleotide sequence (5′-3′)	Application
Upm (long)	CTAATACGACTCACTATAGGGCAAGCAGTGGTATCAACGCAGAGT	RACE PCR
Upm (short)	CTAATACGACTCACTATAGGGC	Universal primers mix
Csp3F	TGYATCATCATYAACAACAAGAA	Conserved region cloning
Csp3R	CATDGASACRATGCADGGGAT	
Csp3A-5-1	AGCTGTGCTGTTTACAG	5′ RACE PCR
Csp3A-5-2	CCTAGCATCAAAGACTGGCTG	
Csp3A-5-3	AAAGTCGAGTGCCACTTTATGG	
Csp3A-3	CGGCTCTTGGTTCATTCAGTCCCTTTGT	3′ RACE PCR
Csp3-E	ATAGAATTCATGAACGGAGACTGCGTG	Recombinant expression
Csp3-X	GAGCTCGAGTCAAGCAGTGAAGTACATCT	
Csp3RTF	GGTTCATTCAGTCCCTTTG	Real-time PCR
Csp3RTR	TACATCTCTTTGGTGAGCAT	
β-actinF	TTCTTGGGTATGGAGTCTTG	Real-time PCR
β-actinR	GTATTTACGCTCAGGTGGG	

### Sequence analysis, multiple sequence alignment and phylogenetic analysis

The nucleotide and deduced amino acid sequences were analyzed using the Expert Protein Analysis System (EXPASY) search program (http://au.expasy.org/tools/). The putative coding sequences (CDSs) were analyzed for the presence of signal peptides using the SignalP 4.0 server (http://www.cbs.dtu.dk/services/SignalP/). A multiple sequence alignment of caspase-3A amino acid sequence of common carp with that of other species was generated using the Clustal W 1.83 program. Homology analysis, including identities and similarities between deduced amino acid sequences of common carp caspase-3A and the other known sequences was performed using MatGat 2.0 [43], [44]. Finally, a phylogenetic tree was constructed using the neighbor joining method within the MEGA software package (version 5.2) and bootstrapped 1000 times. The percentage of the bootstrap values was recorded.

### Carp were treated with Cd

Twenty-four healthy purse red common carp specimens weighing 300±50 g were obtained from a fish hatchery at the Institute of Purse Red Common Carp, Wuyuan County, China. Fish were randomly placed in 6 tanks (4 fish per group) and were kept for 7 days at 12 ± 4°C before experiments. Then, carp were exposed to 2.5 µM Cd [3(CdSO_4_).8H_2_O, groups A and B], 10 µM Cd (groups C and D), 0 µM Cd (groups E and F, as controls). One-third of water in the tank was renewed every 24 h by adding fresh water containing the same concentrations as above.

After 96 h of exposure, the fish groups A, C and E were anesthetized by MS-222 (Sandoz, Switzerland) and sacrificed. Samples of liver tissues were divided into three parts. One part was fixed with cold 4% paraformaldehyde, and the others were weighed and immediately frozen in liquid nitrogen for further processing. Groups B, D and F were treated as groups A, C and E after 168 h of exposure.

### Detection of apoptosis by TUNEL assay

A TUNEL (DNA fragmentation by Terminal deoxynucleotidyl Transferase Biotin-dUTP Nick End Labeling) assay for detection of apoptotic cells was conducted (DeadEnd™ Colorimetric TUNEL System, Promega Corp. USA) as described previously [45]. Finally, TUNEL-positive cells per field were counted in 5 random fields, and the counts were averaged.

### Determination of caspase -3 activity

Caspase-3A activity was measured using caspases-3 Activity Assay Kits (Nanjing Kaiji Biotechnology, China) as previously described [46]. Briefly, liver samples were homogenized in lysate buffer and the homogenates were centrifuged at 15,000 g for 20 min at 4°C. Supernatants were collected and protein concentrations were determined with the Bradford Protein Assay Kit (Beyotime Biotechnology, China). Caspase-3 activity was measured using substrate peptides Ac-DEVD-pNA. The absorbance was measured at a wavelength of 405 nm using a SpectraMax M4 (Thermo Sci.)

### mRNA transcript level analysis of caspase-3A from liver by real-time quantitative PCR

Real-time quantitative PCR was used to analyze the mRNA transcript level of the caspase-3A gene after Cd treatment. Total RNA from liver was extracted using TRIzol reagent (Invitrogen, USA) according to manufacturer’s instructions. The RNA samples were treated with RNase-free DNase (Fermentas, Lithuania) and then 2 µg of the treated RNA was reverse-transcribed with RevertAid^TM^ First Strand cDNA Synthesis Kit (Fermentas, Lithuania). The cDNA fragments of caspase-3A and β-actin were generated by RT-PCR. Amplicons were gel purified, and serial 10-fold dilution was used as a standard curve in each PCR. Real-time quantitative PCR was conducted on a CFX96 Touch™ Real-Time PCR Detection System (Bio-Rad, USA). Amplifications were carried out at a final volume of 20 µl containing 5 µl of 50-fold diluted cDNA template, 10 µl SYBR® Premix Ex Taq™ (Tli RNaseH Plus) (Takara, China), 0.5 µl of each primer (Table 1), and 4 µl ddH_2_O according to the manufacturer’s protocol. PCR amplification was performed in triplicate with the following conditions: 3 min at 94°C, followed by 45 cycles of 10 s at 94°C, 15 s at 55°C and 20 s at 72°C. The reaction which was carried out without cDNA sample was used as a negative control. The results were expressed as the relative fold of the expression of the β-actin gene with the 2^−ΔΔCT^ method [47].

### Production of recombinant proteins and polyclonal antibodies

The complete CDS of caspase-3A was amplified using a primer pair CSP-3E and CSP-3X with *EcoR*I and *Xho*I sites respectively (Table 1). After being digested with the two restriction enzymes, the PCR products were ligated to the PGEX-4T-1 expression vector (Pharmacia Biotech., USA) for constructing recombinant proteins, and then transformed into DH5α-competent cells. After sequencing the positive clones to ensure correct in-frame insertion, the PGEX-CSP3 construct was transformed into *E. coli* BL21 (DE3) strain for prokaryotic expression. The fusion proteins were expressed by isopropyl-beta-D-thiogalactopyranoside (IPTG) induction and analyzed on a 10% SDS-polyacrylamide gel (SDS-PAGE). To prepare the polyclonal antibodies, IPTG was added in a final concentration of 4 mM when the culture reached OD600  =  0.6. After 4 h of culture at 37°C, the cells were harvested by centrifugation and disrupted by sonication. The recombinant proteins were purified by affinity chromatography using a Glutathione Sepharose affinity matrix (GE Healthcare, USA). Recombinant PGEX-CSP3 fusion proteins were eluted from the resin containing 2 M urea, 50 mM KH_2_PO_4_ (pH 7.9), 1 mM EDTA and 50 mM NaCl. The purity of the recombinant proteins was assessed on a 10% SDS-PAGE gel. To generate polyclonal antibodies, we used 4 mg recombinant proteins to immunize rabbits. The obtained polyclonal antibodies were purified using affinity chromatographic column matrix coupled with antigen. The specificity of the polyclonal antibodies was evaluated by Western blot analysis and immunohistochemical detection.

### Western blot analysis

To identify the protein level of caspase-3A in liver, Western blot analysis was performed as we previously described [45], [48]. Briefly, 30 µg of total protein were subjected to 15% SDS-PAGE gel and then transferred electrophoretically onto a 0.45 µm PVDF membrane (Millipore, USA) followed by immunoblotting with the primary antibodies in 1∶500 dilution. Mouse anti-GAPDH monoclonal antibody (Proteintech, China) in 1∶10000 dilution was used as an internal control.

### Immunohistochemical staining of caspase-3A

Immunohistochemical analysis of liver samples was performed as we previously described for kidney tissues [45], [48]. In all analysis, the tissue sections were incubated with diluted (1:100) primary antibodies. Results were evaluated by light microscopy and images were captured by an Olympus BX51 light microscope (Olympus Corp, Japan).

### Statistical methods

The statistical analyses were performed using SPSS software (version 11.5). The frequency of TUNEL-postive cells, caspase-3A activity and its mRNA and protein levels were analyzed by a factorial ANOVA model, represented by 2 independent variables (time, dose) and their interaction. An F-test was done, followed by a Tukey HSD *post hoc* test. Because there were only 2 time periods, *post hoc* test was not required. Statistically significant difference was accepted at *P* < 0.05. All results are expressed as mean ± SD (standard deviation).

## Results

### Molecular characterization and homology analysis of common carp caspase-3A

In this study, we cloned caspase-3 gene of common carp using RACE-PCR method. The full-length cDNA sequence of caspase-3 is 1664-nt (nucleotides) (GenBank accession no. KF055462), containing an 822-nt CDS, a 261-nt 5′ untranslated region (UTR) and a 581-nt 3′ UTR. A putative polyadenylation signal sequence (AATAAA) located at the 18 bp upstream of the poly-A tail. The putative caspase-3 is predicted to encode a peptide of 273 amino acids, with a calculated molecular weight of 30.40 kDa and an isoelectric point of 6.3. No signal petide was predicted by SignalP 4.0 software.

**Figure 1:**
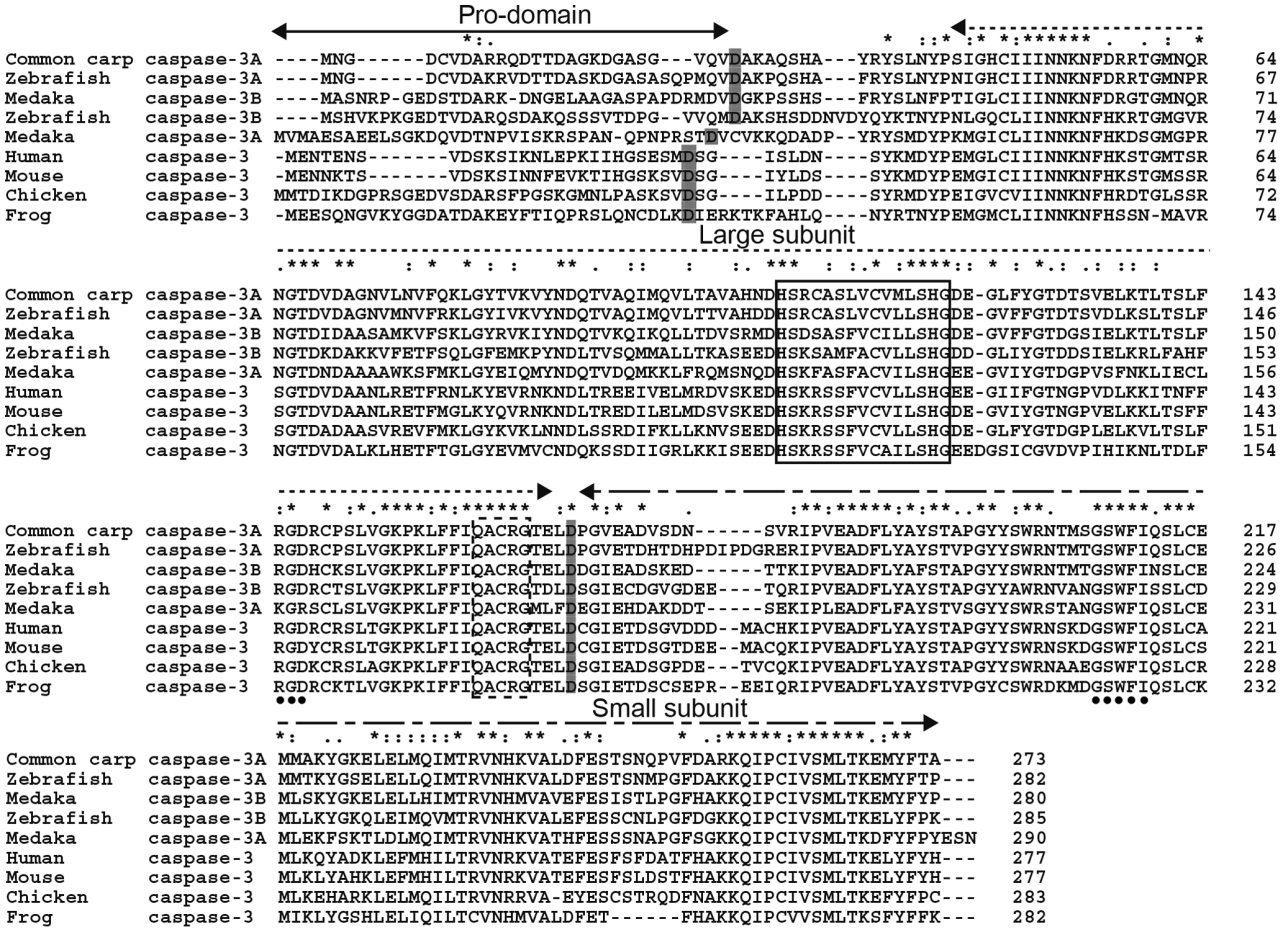
Multiple alignment of caspase-3 amino acid sequences. Identical amino acids are indicated by asterisks, whereas those with high or low similarities are indicated by semicolons and dots. The putative cleavage sites at aspartic acid residues (D), which are shaded in gray, separate the pro-domain (**<—>**), the large subunit (**<·····>**) and the small subunit (**<----->**) respectively. The caspase family signature and the pentapeptide active-site motif (QACRG) were boxed in a continuous and discontinuous line, respectively. The protein binding domain (GSWFI) and integrin recognition motif (RGD) are indicated by solid circles. The GenBank accession numbers of caspase-3 amino acid sequences used here are as follows: zebrafish caspase-3A NP_571952.1, zebrafish caspase-3B NP_001041531.1, Medaka caspase-3A NP_001098140.1, Medaka caspase-3B NP_001098168.1, human NP_116786.1, mouse NP_056548.2, chicken (*Gallus gallus*) NP_990056.1 and frog (*Xenopus laevis*) NP_001081225.1.

The putative caspase-3 presented vertebrate caspase-3 signatures as determined by the InterProScan in the database (http://www.ebi.ac.uk/Tools/pfa/iprscan/). It exhibited a typical caspase-3 domain architecture containing a putative pro-domain (residues 1–26), a caspase family p17 domain (large subunit, residues 43–167) and a p12 domain (small subunit, residues 170–273) (Fig. 1). Furthermore, it contains a characteristic QACQG pentapeptide active-site motif (residues 161–165), which was located in the large subunit, and a caspase family histidine active site HSRCASLVCVMLSHG (caspase family signature, residues 108–122) (Fig. 1). In addition, Asp27 and Asp169 as the common carp putative cleavage aspartic acids separated the pro-domain, the large and small subunits respectively.The integrin recognition motif (RGD, residues 144–146) and the protein binding domain GSWFI (residues 208–212) were conserved. They were located at large and small subunits respectively.

**Figure 2:**
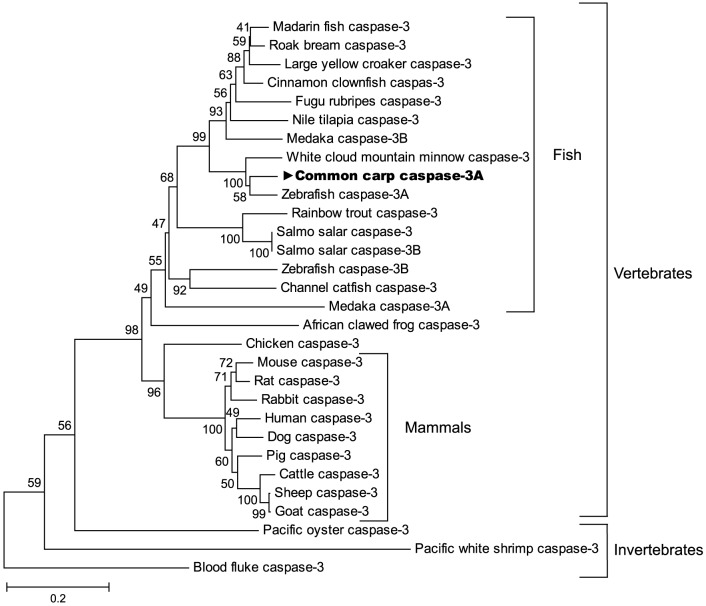
Phylogenetic analysis of common carp caspase-3A. The phylogenetic tree of caspase-3A genes from different species was constructed using the neighbor-joining (NJ) method within MEGA software (version 5.2). The scale bar indicates the average number of amino acid substitutions per site, and the bootstrap values were marked at the nodes of this tree. The GenBank accession numbers of caspase-3 amino acid sequences used here are as follows: rock bream AFM09714.1, large yellow croaker EU878546.1, mandarin fish (*Siniperca chuatsi*) ADK47519.1, cinnamon clownfish (*Amphiprion melanopus*) AEA08874.1, Fugu rubripes (*Takifugu rubripes*) NP_001027871.1, Nile tilapia (*Oreochromis niloticus*) ADJ57601.1, white cloud mountainminnow ACV31395.1, rainbow trout NP_001233264.1, Atlantic salmon NP_001133393.1, Atlantic salmon caspase-3B AAY28972, channel catfish (*Ictalurus punctatus*) NP_001188010.1, rat NP_037054, rabbit (*Oryctolagus cuniculus*) NP_001075586.1, pig (*Sus scrofa*) NP_999296.1, dog (*Canis lupus familiaris*) NP_001003042.1, cattle (*Bos taurus*) NP_001071308.1, sheep (*Ovis aries*) XP_004021739.1, goat (*Capra hircus*) AFC90098.1, blood fluke (*Schistosoma mansoni*) XP_002574296.1 and Pacific white shrimp (*Litopenaeus vannamei*) ABW69658.1. The others are listed in Table 2 and Fig. 1.

Multiple sequence alignment showed that the deduced amino acid sequence of common carp caspase-3 had the highest amino acid identity and similarity to that of zebrafish followed by other fish, then mammals, birds, amphibians and mollusks varying from among 56.4% – 90.4% and 39.7% – 85.8% respectively (Table 2). Because common carp caspase-3 was more identical and similar to zebrafish caspase-3A than its caspase-3B, it was named caspase-3A. In a phylogenetic tree based on amino acid sequences from fish, mammalian, avian, amphibian and invertebrates caspase-3 genes, the caspase-3 proteins from different species were divided into two subgroups (vertebrate and invertebrate). The common carp caspase-3A formed a phylogenetic cluster with cyprinid fish and then other teleost fish, falling finally into the vertebrate subgroup. The topology of the dendrogram is basically in agreement with the concept of traditional taxonomy (Fig. 2).

**Table 2:** Percent identity and similarity of common carp caspase-3A amino acid sequence to other known caspase-3 amino acid sequences.

Species and gene	GenBank accession number	Similarity (%)	Identity (%)
Zebrafish caspase-3A	NP_571952.1	90.4	84.8
Zebrafish caspase-3B	NP_001041531.1	75.4	61.8
Medaka caspase-3A	NP_001098140.1	68.6	52.8
Medaka caspase-3B	NP_001098168.1	80.7	68.9
Atlantic salmon caspase-3	NP_001133393.1	79.2	66.1
Atlantic salmon caspase-3B	AAY28972	79.2	66.1
Human caspase-3	NP_116786.1	71.8	59.3
Chicken caspase-3	NP_990056.1	72.8	58.8
Frog caspase-3	NP_001081225.1	64.5	51.6
Pacific oyster caspase-3	EKC30354.1	57.3	40.7

### Detection of apoptosis by TUNEL assay

**Figure 3:**
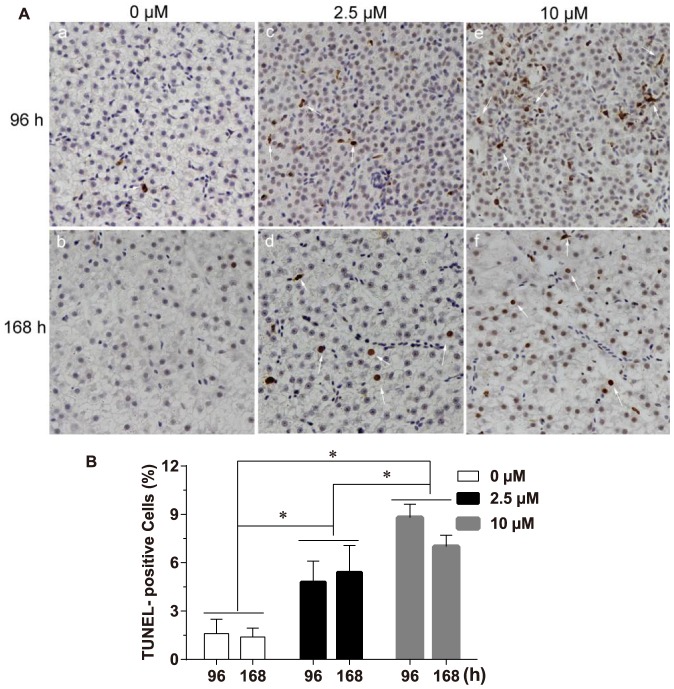
Cd treatment triggered liver apoptosis in purse red common carp. (A) White arrows indicate the brown TUNEL-positive cells (hepatocytes). Representative photographs are shown at 200 × magnification. (B) Quantitative analysis of the percentage of apoptotic cells. Data shown represent mean ± SD from 4 fish in each group, error bars indicate standard deviation. Statistical significance was analyzed using a factorial ANOVA (* *P* < 0. 05).

To assess whether Cd can trigger liver cell apoptosis, TUNEL assay was used to identify the extent of DNA fragmentation. Few single apoptotic cells were detected in the control groups [Fig. 3A(a and b)]. Whereas more apoptotic cells were present in Cd-treated groups [Fig. 3A(c-f)]. TUNEL-positive staining was mainly focal on hepatocytes, and the nuclei of the apoptotic cells were stained into brown. Statistical analysis showed that the percentage of apoptotic cells was enhanced in liver after Cd treatment (*F*  =  90.824, *P* < 0.05). The following Tukey HSD test indicated that the percentage of apoptotic cells in liver after exposure to different concentrations of Cd followed the hierarchical pattern: groups exposed to 10 µM Cd > groups exposed to 2.5 µM Cd > control groups (*P* < 0.05) (Fig. 3B). In contrast, Cd exposure time did not significantly influence the frequency of apoptotic cells (*F*  =  1.441, *P* > 0.05). In addition, no significant interaction between the two factors (time and concentration) was detected (*F*  =  3.294, *P* > 0.05).

### Determination of caspase-3A activity

**Figure 4:**
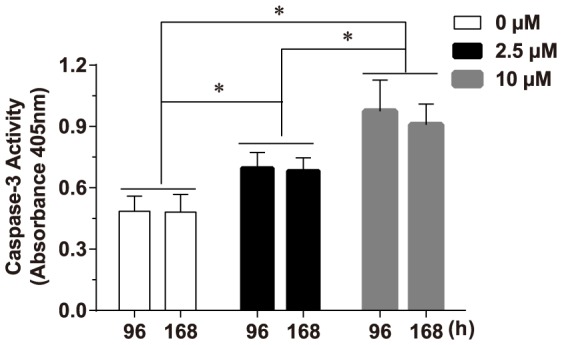
Effects of Cd treatment on caspase-3A activity in liver from purse red common carp. The extracts were incubated with the specific substrates of caspase-3 and release of *p*-nitroanilide was measured at 405 nm. Values represent the mean ± SD; error bars indicate standard deviation. Statistical significance was analyzed using a factorial ANOVA (* *P* < 0. 05).

To consolidate whether Cd could activate caspase-3A, caspase-3A activity was measured. The result showed that caspase-3A activity was significantly enhanced in liver after Cd exposure (*F*  =  45.213, *P* < 0.05). The following Tukey HSD test revealed that caspase-3 activities in different groups exhibited the following hierarchical pattern: groups exposed to 10 µM Cd > group exposed to 2.5 µM Cd > control groups (*P* < 0.05) (Fig. 4). Whereas caspase-3 activity was not affected by exposure time (*F*  =  0.517, *P* > 0.05), and there was not significant interaction between the two factors (time and concentration) (*F*  =  0.237, *P* > 0.05).

### Changes of caspase-3A mRNA level upon Cd challenges

**Figure 5:**
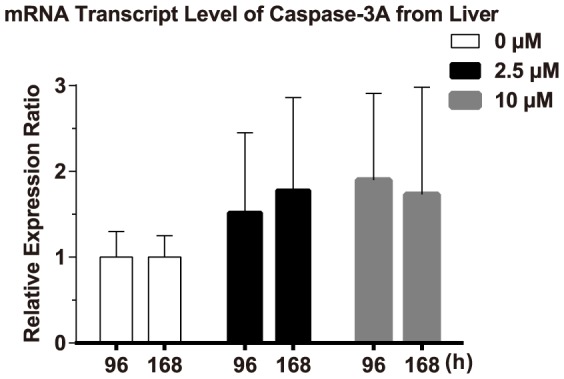
Effect of Cd treatment on mRNA transcript level of caspase-3A. The changes of mRNA levels were determined by real-time quantitative PCR. β-actin was used as a reference gene to normalize caspase-3A. The relative expression ratio was calculated with the 2^−ΔΔCT^ method. Error bars indicate standard deviation. Values represent the mean ± SD. of 4 independent samples. Statistical significance (*P* < 0.05) was analyzed using a factorial ANOVA. The mRNA levels of caspase-3A in carp were not significantly increased after exposure to Cd (*P* > 0.05).

Real-time quantitative PCR was used to analyze the mRNA transcript level of caspase-3A in the liver. Fish exposed to 10 µM Cd for 96 h exhibited a maximum relative mRNA level, which was about 1.90-fold of the control group (Fig. 5). But according to the factorial ANOVA analysis, the mRNA level of caspase-3A was not significantly affected by exposure time (*F*  =  0.094, *P* > 0.05) or exposure concentration (*F*  =  2.226, *P* > 0.05). Meanwhile, the interaction between the two factors (time and concentration) was also not significant (*F*  =  0.031, *P* > 0.05).

### Production of fusion proteins and immunodetection of caspase-3A by Western blot analysis

**Figure 6:**
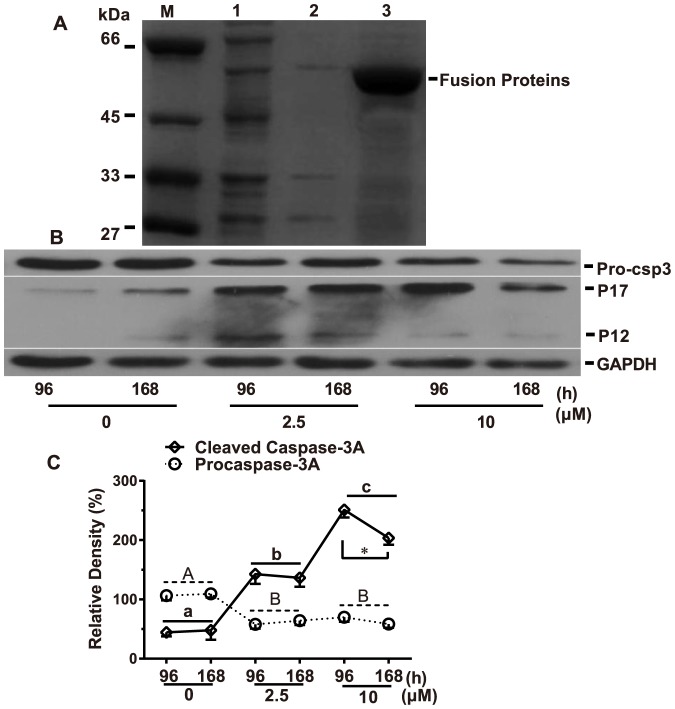
Dissolvability analyses of fusion protein and protein expression analysis (A): common carp caspase-3A clones expressed in *E. coli.* Lane M: protein molecular standard; Lane 1: total cellular proteins of PGEX-GST/BL21 after induction; Lane 2: supernate of purified recombinant PGEX-CSP3A; Lane 3: precipitate of purified recombinant PGEX-CSP3A. (B): Western blots showing procaspase-3A/ (Pro-csp3) and cleaved fragments (p17 and p12) after Cd treatment. (C): Protein band density was analyzed with the Gel-Pro Analyzer. GAPDH was used as a loading control. Each value was expressed as the ratio of caspase-3A proenzyme and its activated form (the sum of expression levels of p17 and p12 forms) to GAPDH level, which represents the mean ± SD. of 4 independent samples performed in triplicate. Statistical analysis was performed using a factorial ANOVA. Different upper case letters represent significant differences of proenzyme levels between exposure concentrations (*P* < 0.05), while different lower case letters represent significant differences of activated forms between exposure concentrations (*P* < 0.05) and asterisk (*) shows the significant difference between the two exposure time points for the same Cd-induced concentration (*P* < 0.05)

Recombinant capase-3A was produced in *E. coli* BL21 (DE3) cells after being cloned into the pGEX-4T-1 expression vector. After IPTG induction, the pGEX-4T-CSP3 fusion proteins, which were produced and mainly expressed in precipitate (Fig. 6A), were used to immunize rabbits and the produced primary antibodies were detected by Western blot analysis.

To elucidate whether caspase-3A activation is involved in Cd-induced liver cell apoptosis, time and concentration course experiments were carried out by analyzing the activation status of caspase-3A using Western blot analysis. Changes of caspase-3A cleavage were used as a marker for the activation of caspase-dependent pathway. In mammals and zebrafish, caspase-3 exists as a proenzyme (procaspase-3), which is about 32 kDa [34], [49]. It can be proteolytically cleaved to form 17 kDa (p17) and 12 kDa (p12) fragments. The results confirmed the proteolytic cleavage of caspase-3A with Cd treatment in a concentration-dependent manner [Fig. 6(B and C)]. The reduction of 32 kDa proenzyme (p32, procaspase-3A) was accompanied by a concomitant increase in the cleaved p17 and p12 fragments, reaching maximal levels in groups D (exposure to 10 µM Cd for 168 h). Meanwhile, the increases of activated caspase-3A (p17 and p12) peaked in groups C (exposure to 10 µM Cd for 96 h).

The factorial ANOVA analysis showed that the proenzyme form was markedly affected by exposure concentrations (*F*  =  110.183, *P* < 0.05) but not by exposure time (*F*  =  0.049, *P* > 0.05). The following Tukey HSD test indicated that the protein level of proenzyme in different Cd treatment conditions followed the hierarchical pattern: groups exposed to 10 µM Cd and groups exposed to 2.5 µM Cd < control groups (*P* < 0.05). Whereas the protein levels of cleaved forms were significantly influenced not only by exposure time *(F*  =  357.264, *P* < 0.05), but also by exposure concentrations (*F*  =  9.183, *P* < 0.05). The interaction between the two factors (time and dose) about the protein levels of cleaved forms was significant (*F*  =  7.958, *P* < 0.05). The further pairwise comparisons showed that only exposure to 10 µM Cd resulted in different expression level of cleaved forms between the two time points (Fig. 6C). The Tukey HSD test indicated that the protein levels of cleaved forms in groups treated with different concentrations of Cd followed the hierarchical pattern: groups exposed to 10 µM Cd > groups exposed to 2.5 µM Cd > control groups (*P* < 0.05) (Fig. 6C). Overall, these results indicate that Cd can effectively activate caspase-3A.

### Immunohistological localization of caspase-3A in liver

**Figure 7:**
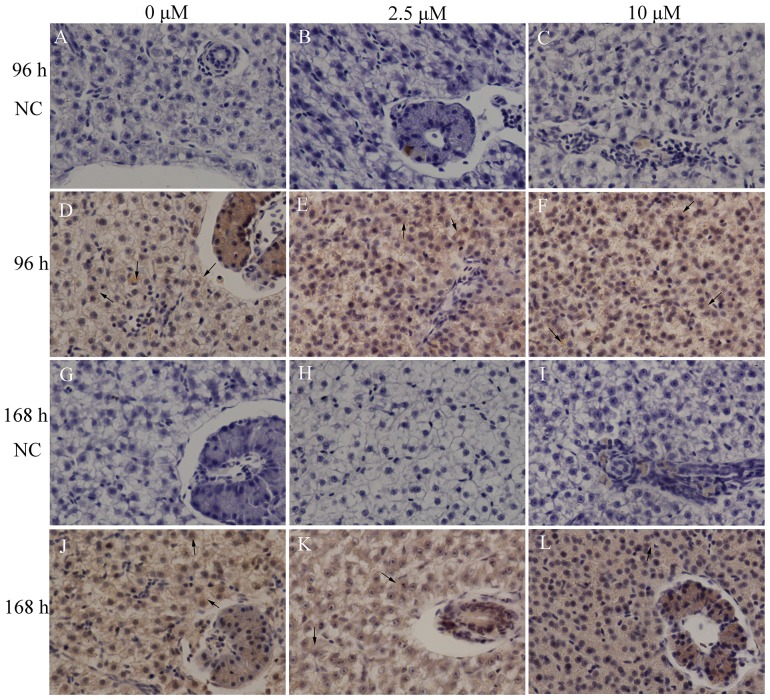
Immunolocalization of caspase-3A in liver sections. NC represents negative control (substitution normal non-immune serum from the same host animal as the primary antibody). All the negative controls show no positive staining [Fig. 7(A-C), 7(G-I)]. Both control groups [Fig 7(D and J)] and experimental groups [Fig 7(E, F, K and L)] reveal weak immunolabeling. Arrows indicate the cytoplasm of hepatocytes. The representative photographs are shown at 200 × magnification.

Results for negative controls (substitution normal non-immune serum from the same host animal as the primary antibody) showed that no positive reaction was observed [Fig. 7(A-C and G-I)]. The immunoreactivity of caspase-3 was observed focally in the cytoplasm of hepatocytes in all groups [Fig. 7(D-F and J-L)]. The immunostaining intensities in Cd-exposed groups [Fig. 7(E, F, K and L)] were similar to those in control groups [(Fig. 7(D and J)] with weak immunoreactions.

## Discussion

Caspase 3 is a crucial molecule in regulating both mitochondrial and death receptor apoptotic pathways [50]. It has a conserved apoptosis-related function in fish and mammals [31]–[36], [41], [51], so a similar role that cleaves the majority of the caspase substrates is also to be expected in fish. Previous reports show that two genes homologous (caspase-3A/-3B) to mammalian caspase-3 have been identified in zebrafish, Atlantic salmon and Medaka. However, the physiological role of these two isoforms has not been illustrated [52]. In this study, only one caspase-3 isoform was found and characterized. The deduced amino acid sequence of common carp caspase-3A was highly homologous with those previously reported caspase-3 genes. It exhibited the typical characteristics of known caspase-3 genes with a putative pro-domain, large and small subunits, a pentapeptide active-site motif, cysteine active sites, etc. The similarity of caspase-3A structure between common carp and other vertebrates suggests that the common carp caspase-3A may have conservative functions as vertebrates have. Phylogenetic analysis also showed that common carp caspase-3A was classified with known caspase-3 subgroup of fish, which further indicates that caspase-3A may be functional analogues of mammalian caspase-3 genes.

After exposure to Cd, TUNEL analysis showed the percentage of apoptotic cells in liver was significantly elevated in a dose-dependent manner. It reveals that Cd can effectively induce liver cell apoptosis in common carp. Furthermore, caspase-3 activity was also markedly increased in a dose-dependent manner. It indicates Cd can activate caspase-3A. In mammals, caspase-3 proenzyme exists within the cytosol as inactive dimers. Once activated, it can cleave vital intracellular proteins and controls various signal transduction pathways [53]. In this study, the activation of caspase-3A was further supported by the Western blot analysis, which showed that the protein level of procaspase-3A was decreased, whereas the cleaved form of caspase-3A was increased. Both of the alterations of proenzyme level and cleaved forms occurred in a dose-dependent manner. The results are agreement with our previous reports, where Cd induces kidney apoptosis with caspase-9 activation [45], [48]. The activation of capase-9 can result in the activation of effector caspases such as caspase-3 and -7. It implies that the Cd-induced liver cell apoptosis of common carp through the activation of caspase-3A. The findings are consistent with other studies performed in various cell types and tissues, reporting an involvement of caspase-dependent pathway in the Cd-induced apoptosis [26], [54]–[56]. However, in this study, exposure time did not significantly influence the process of apoptosis totally. The possible reason is that the time interval between the two exposure time points is short.

Previous studies have shown that other toxicants such as copper [57], aluminum [58], [59], fluoride [60] and Anatoxin-a [61], or prolonged hypoxia [62] can activate caspase-3 or trigger apoptosis in common carp. When compared to aluminum [58], Cd seems to exhibit a weak effect in activating caspase-3. The difference could be caused by different experimental conditions such as water temperature, exposure concentration and duration, fish size and assay kits besides their special characters. It is important to consider that both the exposure conditions and the experimental model (e.g. cell types and animals) can affect apoptotic progress and even cause different cell death mechanisms. Furthermore, numerous studies have confirmed that Cd can mediate various signaling pathways that trigger caspase-dependent or caspase-independent apoptosis [63]. In this model of Cd-induced liver cell apoptosis in common carp, Cd may possibly induce multiple signaling pathways. That detecting more signaling molecules such as Fas/ Fas Ligand, bax and bcl-2 is necessary to clarify the Cd-induced apoptotic pathway and explore the role of caspase-3A.

In fish, the mRNA transcript levels of caspases are usually used as a marker at an earlier point in the apoptotic cascade. Some reports have shown that caspases mRNA levels are increased in fish after heavy metal or other apoptotic stimuli treatment [16], [18]. However, the mRNA transcript level of caspase-3A did not differ significantly between Cd-treated groups and control groups in our study. In fact, it is rare to detect the occurrence of apoptosis by determining caspases mRNA levels in mammals [64]. The mRNA levels of caspases are only thought to reflect the amount of procaspases. It cannot reflect the level of biologically active caspases [65]. This is the possible reason why the mRNA levels of caspase-3A did not alter after Cd treatment. It is necessary to employ two or more distinct assays to confirm that cell death is occurring via apoptosis [66].

Although the immunohistochemical analysis does not accurately evaluate the intensity of protein expression, our result still showed that the expression levels of caspase-3A remained relatively stable in liver after Cd exposure. The caspase-3A immunoreactivity was mostly found in the cytoplasm. An early report indicates that Cd and copper induce a marked increase of caspase-3 positive cells of head kidney hematopoietic tissue in common carp [67]. However, in that report, a commercially available primary antibody (for mammals) that recognizes cleaved or activated caspase-3 was used. The result reflects the expression changes of activated forms. In our study, the polyclonal rabbit antibody that we produced detected procaspase-3A and their cleaved fragments at the same time. The immunoreactivity was possibly the mixed effect of caspase-3A proenzyme form and its activated forms. Our findings agree with a previous report, which shows that immunostaining of caspase-3 is predominantly cytoplasmic and no noticeable changes of immunoreactivity is observed either in the Cd-exposed group or in its control group [68]. Because caspase-3 activation is associated with the occurrence of apoptosis [69], it is necessary to use primary antibodies that can detect cleaved forms when we evaluate caspase-3 immunohistochemical expression.

## Conclusions

In this study, the full-length cDNA sequence of caspase-3A gene was isolated from the liver tissue of common carp. The cloned caspase-3A gene is homologous to that of other vertebrates. The general results of increased frequency of apoptotic cells and caspase-3A activity, significantly decreased precursor protein and the noticeable increased activated forms of caspase-3A show that Cd can induce liver cell apoptosis through caspase-3 activation in common carp. The mRNA level of caspase-3A did not vary after Cd exposure, which reveals the mRNA level cannot be used as the major marker at an earlier point in the apoptotic progress. Meanwhile, immunopositive staining was mainly detected in the cytoplasm of hepatocytes. The detailed regulating mechanism and other possible signaling pathways remain to be further studied.
